# BioBone – A prospective, blinded, multicenter validation study of the CD8 + terminal differentiated effector memory cells (CD8 + TEMRA cells) as prognostic biomarker for disturbed fracture healing – study design

**DOI:** 10.1186/s13018-025-05987-7

**Published:** 2025-06-16

**Authors:** Simon Reinke, Anja Maria Bauer, Michael Dahne, Georg Duda, Denis Gümbel, Christian Kleber, Georg Matziolis, Sven Märdian, Georg Osterhoff, Carsten Perka, Michael J. Raschke, Sebastian Rohe, Klaus-Dieter Schaser, Philipp Schwabe, Frederik Maximilian Schäfer, Richard Stange, Maik Stiehler, Ulrich Stöckle, Hans-Dieter Volk, Stefan Weber, Sven Geißler, the BioBone Author Consortium, Martin Adelmann, Doruk Akgün, Levent Akyüz, David A. Back, Antje Blankenstein, Karl F. Braun, Matthias Bungartz, Andreas Böhmler, Ioanna Maria Dimitriou, Stefanie Donner, Christian Eder, Axel Ekkernkamp, Tilmann Engelhardt, Frank Graef, Gerald Grütz, Lea Heinemann, Johannes Herold, Christoph-Eckhard Heyde, Christian Hipfl, Andreas Hüser, Suchung Kim, Mario Koksch, Kirsten Labbus, Luis Lauterbach, Sarah Marie Litschel, Cornelia Lützner, Tazio Maleitzke, Elli Mann, Daniela Nagel, Marcel Niemann, Melanie Ort, Alessandra Penaverde, Johanna Penzlin, Simone Preck, Wera Pustlauk, Franziska Radach, Marie Reisener, Jana Riecke, Gabriele Rußow, Stephan Schlickeiser, Friederike Schömig, Anne Schützer, Firas Souleiman, Nina Stelzer, Dirk Stengel, Matthias Streitz, Martin Textor, Serafeim Tsitsilonis, Tu Lan Vu-Han, Eric Jörg Walther, Tobias Winkler, Silvan Wittenberg, Regina Zappel

**Affiliations:** 1 Berlin Institute of Health Center for Regenerative Therapies, Charité – Universitätsmedizin Berlin, Berlin, Germany; 2 Berlin Institute of Health at Charité – Universitätsmedizin Berlin, Julius Wolff Institute, Berlin, Germany; 3 Charité – Universitätsmedizin Berlin, corporate member of Freie Universität Berlin and Humboldt-Universität Zu Berlin, Center for Musculoskeletal Surgery, Berlin, Germany; 4 UniversitätsCentrum Für OrthopädieUnfall- Und Plastische Chirurgie (OUPC), Universitätsklinikum Carl Gustav Carus an Der Technischen Universität, Dresden, Germany; 5 Department of Regenerative Musculoskeletal Medicine, Institute for Musculoskeletal Medicine (IMM), University Hospital Münster, Münster, Germany; 6 Department for Trauma and Orthopaedic Surgery, Vivantes-Hospital Spandau, Berlin, Germany; 7 Department for Orthopaedics, Trauma and Plastic Surgery, Leipzig University Hospital, Leipzig, Germany; 8 Orthopaedics University Hospital Jena, Campus Eisenberg, Friedrich-Schiller-University Jena, Eisenberg, Germany; 9 Trauma Surgery and Orthopedics Clinic, BG Hospital Unfallkrankenhaus Berlin, Berlin, Germany; 10 Charité - Universitätsmedizin Berlin, corporate member of Freie Universität Berlin and Humboldt Universität Zu Berlin, Institute of Medical Immunology, Berlin, Germany; 11 CheckImmune GmbH, Berlin, Germany; 12 Beckman Coulter Life Sciences, Krefeld, Germany; 13 Berlin Institute of Health, Berlin-Brandenburg School for Regenerative Therapies, Charité – Universitätsmedizin Berlin, Berlin, Germany; 14 Department of Radiology, Charité - Universitätsmedizin Berlin, Corporate Member of Freie Universität Berlin and Humboldt-Universität Zu Berlin, Berlin, Germany; 15 Clinic of Trauma, Hand and Reconstructive Surgery, University of Rostock, Rostock, Germany; 16 Department of Experimental Animal Facilities and Biorisk Management, Friedrich-Loeffler-Institut, Greifswald, Germany; 17 BG Kliniken - Hospital Group of the German Federal Statutory Accident Insurance, Berlin, Germany; 18 Department of Trauma, Hand and Reconstructive Surgery, University Hospital Münster, Münster, Germany; 19 Department of Plastic and Hand Surgery, Burn Centre, BG Klinikum Bergmannstrost Halle, Halle, Germany

**Keywords:** Long bone fracture, Impaired healing, CD8 + TEMRA, Prognostic biomarker

## Abstract

**Aims:**

The BioBone consortium aims to validate circulating CD8 + TEMRA cells as a prognostic biomarker for predicting impaired fracture healing outcomes in a prospective, blinded, multicenter clinical study. The primary performance parameters are the pre-operative identification of at least 30% of patients who ultimately experience impaired healing at the first clinical endpoint, with a specificity greater than 90% to minimize the false-positive rate.

**Methods:**

BioBone is a prospective, blinded, multicenter biomarker validation study designed to assess the prognostic value of circulating CD8 + TEMRA cells in fracture healing. A total of 640 patients aged 18 to 80 years with fractures of the humeral diaphysis, radial and/or ulnar diaphysis, femoral neck, trochanteric femur, femoral diaphysis, distal femur, proximal tibia, tibial diaphysis and distal tibia will be enrolled. The study is powered to validate the target assay performance and accounting for 6–7 potential confounders at an expected incidence of 10% impaired healing. Biomarker levels will be measured pre- and post-operatively using flow cytometry (FC) and patients will be monitored for one year. The primary endpoint is fracture healing status at 17–19 weeks (normal healing or delayed healing), while the secondary endpoint evaluates healing at nine months (delayed healing or pseudarthrosis). Fracture consolidation will be assessed through radiographs or computed tomography (CT) scans in conjunction with clinical assessments such as range of motion and weight-bearing capacity. Key outcome measures include radiographic analysis (RUST/RUSH scores), functional and patient-reported outcomes (e.g. weight bearing ability, range of motion, and the SF-36 questionnaire), as well as socioeconomic parameters (e.g. work capacity, rehabilitation needs, mobility). The predictive performance (sensitivity, specificity, NPV, PPV) of the biomarker will be determined in a prospective, double-blinded analysis, where CD8 + TEMRA blood levels are measured prior to surgical treatment and healing status at clinical endpoints is assessed by independent observers. Additional immunological examination and in vitro analysis of blood and fracture hematoma samples will further investigate the mechanism of action of CD8 + TEMRA cells in impaired human bone regeneration.

**Conclusion:**

The BioBone study will validate the suitability of CD8 + TEMRA cells as a prognostic marker for impaired fracture healing and their integration into routine clinical practice. The results could have a global impact by incorporating immune-based prognostic tools into clinical workflows, paving the way for precision medicine approaches in trauma care. The BioBone study is funded by the German Federal Ministry of Education and Research (BMBF).

## Introduction

In 2019, 178 million bone fractures were reported worldwide, most commonly humeral, radial, tibial and femoral fractures [1]. Although bone has a very high endogenous regenerative potential, the prevalence of non-union or delayed fracture healing ranges between 5 and 15% in industrialized nations [2–4]. These patients require additional surgical interventions (median of 2 revision procedures [5–7], leading to prolonged hospitalization and rehabilitation periods (median time = 23 months [7]. Health economic analyses suggest that in-patient costs alone range from 18,000–25,000€ per compromised healing case, although this varies depending on the localization of the fracture, the type of treatment, and the country-specific health care system [8]. When out-patient treatment and productivity losses are included, total costs increase to 36,800–50,000€ per case, underscoring the socioeconomic challenge [9, 10].

Currently there are no reliable prognostic methods for early prediction of patients at risk of compromised fracture healing outcomes [11–13]. Hence, there is a clear medical demand for novel approaches enabling early patient stratification to guide therapeutic strategies and improve bone healing. Such strategies include advanced surgical interventions [14, 15], tailored rehabilitation programs or fixation devices, which are currently only applied in revision surgery. Early prediction of the risk for compromised healing would not only reduce the financial burden associated with prolonged care and lost productivity, but also improve the quality of life of affected patients through timely and personalized interventions [12, 16–18].

Recent evidence highlights the critical role of adaptive immunity in bone repair, with T cells modulating healing even in the absence of infection [19–22]. Delayed fracture healing correlates with increased levels of CD8 + terminally differentiated effector memory T cells (CD8 + TEMRA) [23–25]. This difference is long-lasting and reflects an individual immune profile rather than an acute immune response to injury. The frequency of CD8 + (CD3 + CD8 + CD28-CD57 +) TEMRA cells is determined by lifelong antigen exposure and correlates with the overall immune experience. Furthermore, they proliferate poorly but have potent effector functions such as cytotoxicity and cytokine release. CD8 + TEMRA cells can be directly activated by stress signals or pro-inflammatory cytokines without antigen presentation. These cells are enriched in the fracture hematoma where they act as major producers of pro-inflammatory cytokines such as interferon-γ and tumor necrosis factor-α [26]. This disrupts the resolution of the initial inflammatory healing phase, impairs pro-osteogenic cell function and inhibits osteogenesis. In animal studies, adoptive (systemic) transfer of CD8 + T cells completely impaired bone healing, whereas antibody-mediated depletion of circulating CD8 + T cells significantly enhanced endogenous fracture repair. These findings highlight the prognostic potential of CD8 + TEMRA cell quantification for predicting fracture healing outcomes and could assist to adapt the treatment strategy [23, 27, 28].

### Aims and Objectives

The aim is to conduct a prospective, blinded clinical validation study of the CD8 + TEMRA cells as a prognostic biomarker to predict healing outcome after fracture. Aimed test characteristics are a positive predictive value (PPV) > 60% and a specificity > 90%, as well as the clinical routine suitability or cost-effectiveness. We hypothesize that:

1. The perioperative cut-off level of CD8 + TEMRA cells (> 38%) is a prognostic marker with a high diagnostic accuracy to distinguish between patients with normal-, delayed or incomplete (non-union) fracture healing.
2. Poor bone fracture healing can be predicted with a very high specificity (> 90%) prior to surgical intervention, allowing a more personalized treatment to improve the outcome with high cost effectiveness.
3. As the biomarker reflects the pathogenic impact of TEMRA cells on fracture healing, precise identification of poor healers would offer novel immune-modulatory approaches to improve poor healing.

## Methods

### Ethical considerations

The CD8 + TEMRA cells will be analyzed in EDTA blood samples of < 2 ml, which are usually taken in addition to routine blood collection at the admission of the patients for the surgery intervention. There is no additional risk for the patients and the study has no influence on standard care of the patients. The data are protected according to the regulatory rules and the participants’ confidentiality is guaranteed. The blood collection for the validation as well as the study procedures are already approved by the local institutional review board (IRB) of the Charité for the recruitment center Charité Universitätsmedizin Berlin, Unfallkrankenhaus Berlin, Vivantes Klinikum Spandau (IRB number: EA2/096/11), University Hospital Carl Gustav Carus (IRB number: 310072016), University Hospital Münster (IRB number: 2018–728-b-S), Friedrich-Schiller University, Jena (IRB number: 4689–02/16) and University Hospital Leipzig (IRB number: 299/18-lk).

#### Inclusion–exclusion criteria

The patients will be recruited at the following clinical recruitment centers: Charité Universitätsmedizin Berlin, Unfallkrankenhaus Berlin, Vivantes Klinikum Spandau, University Hospital Leipzig, Waldkliniken Eisenberg, University Hospital Münster and University Hospital Carl Gustav Carus Dresden.

Adhering to the following inclusion and exclusion criteria, we will perform a compatibility assessment and all eligible patients will be informed about the study content to receive their consent. Written consent will be obtained from each subject to be involved in the clinical study by using the IRB approved informed consent form prior to the conduct of any study-related activity. The subjects will also be instructed that their participation is voluntary and that they are free to withdraw their consent and to discontinue their participation at any time without prejudice (Table 1).

**Table 1:** Inclusion–exclusion criteria

**Inclusion Criteria:** Subjects must meet all of the inclusion criteria listed below to be eligible for the study
1. Male or female subjects 2. Subjects 18—80 years of age at the time of screening 3. Only closed fractures 4. Fractures of the humeral diaphysis, radial and/or ulnar diaphysis, femur or tibia 5. Monotrauma or type of trauma with comparable post-surgery mobilization regime 6. Osteosynthesis 7. Signed informed consent form
**Exclusion Criteria:** Subjects with any one of the exclusion criteria listed below will not be eligible for the study
1. Cancer related fracture 2. Periprosthetic fracture 3. Additional long bone fracture with the exception of index fractures (inclusion criteria) 4. Additional thoracic trauma 5. Known active hepatitis B virus or hepatitis C virus infection at screening 6. Known human immunodeficiency virus (HIV) infection, severe uncontrolled inflammatory disease or severe uncontrolled autoimmune disease (e.g., ulcerative colitis, Crohn’s disease, etc.) 7. Active malignancy or history of malignancy within 5 years prior to screening 8. Known diagnosis of moderate to severe dementia based on subject’s medical history or severe psychiatric disorder 9. Known history of drug or alcohol abuse in the past 12 months, based on self-report or medical record 10. History of autologous/allogeneic bone marrow transplantation 11. Exposure to allogeneic cell-based therapy in the past or exposure to autologous cell therapy in the last 12 months before screening 12. Pregnancy 13. Subject is currently enrolled in an investigational device or drug trial, or has not yet completed a period of at least 30 days since ending other investigational device or drug trial(s) 14. Subject is detained or institutionalized under a court order or administrative order 15. In the opinion of the investigator, the subject is unsuitable for participating in the study (patient compliance)

#### Sample size calculation

A total number of 640 patients must be completely analyzed. This number is required to simultaneously construct a 97.5%-confidence interval with half-length of 3% for an expected specificity of 90% and a 97.5%-confidence interval with a half-length of 10% for an expected sensitivity of 85% associated with the assumed cut-off and a prevalence of delayed fracture healing of approximately 10%. The 97.5%-confidence intervals are used to maintain a global alpha error of 5%. Assuming a consent rate of 70% among patients meeting all inclusion and exclusion criteria and a dropout rate of 20%, approximately 1,140 patients will need to be screened to enroll 800 participants and obtain 640 complete datasets for analysis. Sample size calculation was done as described by Krummenauer and Kauczor [29]. According to this sample size, we use Harrell's rule that 10 to 20 events per independent predictor variable are necessary to ensure a stable regression model. With the above sample size, approximately 6 predictor variables, respective covariables or confounders 640*0.1/10 can be entered in a final logistic regression model to discriminate regarding the fracture healing outcome [30].

### Blood sample collection and biomarker assay

The biomarker CD8⁺-TEMRA cells is analyzed in EDTA-anticoagulated blood samples (up to 2 ml), which are taken in addition to the routine blood tests on admission of the patient (before surgery). All samples are processed and analyzed centrally at the BIH Center for Regenerative Therapies in Berlin to ensure uniform methodology and quality control at all sites. To ensure the integrity of biomarker measurement, blood samples must meet standard pre-analytical conditions, including avoidance of clotting and hemolysis. Study teams at all recruiting centers receive specific training on proper sample handling, focusing on critical factors such as proper fill volume, gentle mixing (to avoid shaking), protection from direct sunlight and appropriate temperature control. The transportation logistics are established and ensure that biomarker measurements can be performed within 24 h of blood collection. It includes two transportation scenarios: Scenario 1) For samples collected between 10:00 am and 5:00 pm, DHL Express is used to ensure delivery to the Berlin lab by 9:00 am the next day. Scenario 2) For samples taken between 17:00 and 22:00 (e.g. from patients requiring urgent surgery), the DHL Same Day Service is used. Samples from the Berlin recruitment centres (Vivantes Klinikum Spandau and Unfallkrankenhaus Berlin) are transported via the Labor Berlin – Charité Vivantes GmbH shuttle system. Upon receipt at the central laboratory, the CD8⁺ TEMRA biomarker (defined as CD45⁺CD3⁺CD8⁺CD57⁺CD28⁻) is determined using the Beckman Coulter DuraClone IM T Cell Subset Tube (Panel B53328), following the manufacturer's protocol. Flow cytometric data are collected and analyzed using Beckman Coulter's Kaluza software. This standardized workflow has been validated in previous studies on different patient groups and demonstrates robust assay performance, with coefficients of variation (CVs) below 10% [25, 31–33].

### Study design

The study is designed as a phase II-III, prospective, multicenter, blinded, national validation study in a realistic (“routine-adapted”) clinical setting. Thus, all study visits are part of the clinical routine, including the radiological and clinical evaluation.

**Fig. 1:**
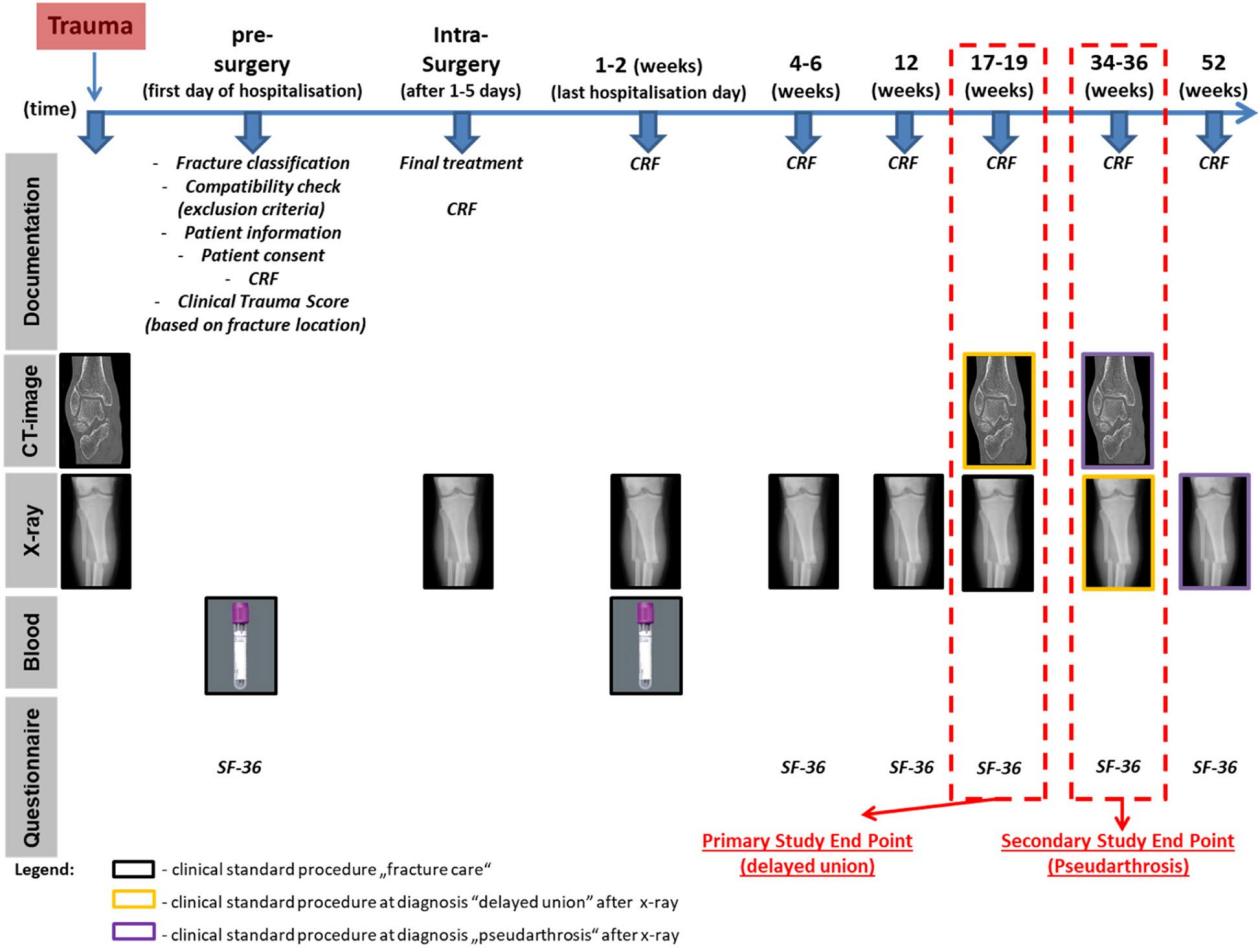
Study Design. At the primary study end point (17–19 weeks), the patients will be classified as normal or delayed healing patients. The latter undergo radiographic/CT analysis at the secondary study endpoint (34–36 weeks) in order to subgroup them into delayed or non-union patients. At the final study time-point (52 weeks), the healing outcome of the non-union will be evaluated via radiographic/CT analysis. Functionality tests and the Short Form 36 Health Survey (SF-36) assessment will be performed with all patients at all study time-points

A total of 7 clinical level 1/2 hospitals in Germany are included in the study. To ensure the prospective clinical validation of the CD8 + TEMRA cells as a prognostic marker for disturbed fracture healing, a monitoring of the complete fracture regeneration process is required. Therefore, we calculated a total study time of 52 weeks including 8 clinical visits (Fig. 1).

The initial radiological assessment will be used for the fracture classification and, in addition to the clinical and laboratory findings, the selection of the best surgery intervention method. Adhering to the inclusion and exclusion criteria, we will perform a compatibility assessment, and all eligible patients will be informed about the study content prior to receiving their consent. Blood samples for CD8 + TEMRA cell expression will be taken during the patient´s inhouse admittance prior the surgical intervention. Healing progress will be monitored via clinical/radiological assessment during the following clinical visits. The SF-36 questionnaire will be used to assess the patient’s quality of life. All patient data will be documented via secuTrial software in the electronical case report form (eCRF). Based on healing status of the patients at the primary study end point, subjects will be classified as normal or delayed healers. Normal healing patients will only perform the clinical/functionality tests and SF-36 at the following visits. At the secondary end point, delayed healing patients will be classified further as delayed or non-union according to additional radiographic and clinical evaluation. Whereas the delayed healing patients only perform clinical/functionality tests and SF-36 at the following visit, the non-union patients undergo a final radiographic evaluation at week 52 post injury.

### Data collection

Within the study, the following parameters are recorded and entered into the secuTrial system via electronic case report form (eCRF). The software is compliant to FDA CFR 21 part 11 and, thanks to its web-based design, can be accessed worldwide at any time using any standard internet browser. By this all requirements for the use in studies will be in accordance with the German Drug Law (Arzneimittelgesetz AMG) (Table 2).

**Table 2:** Clinical, socio-economic and health data collection of the BioBone study

**Basic data (FU visits 1–3)**	
Date of fracture, date of hospital admission, date of surgery, date of hospital discharge, accident mechanism (vehicle occupant, two-wheeler rider, pedestrian, fall, sports), work accident, age, sex, height, weight, profession	
**Life Style (FU visits 1–3)**	
Smoking status	Smoker, previous smoker, non-smoker
Alcohol consumption	Units/week
Sporting activities	Activity type, h/week,
**Surgery and fracture related information (FU visits 1–3)**	
Number of fractures	1 or ≤ 2
ASA score	I—IV
AO classification	Femur, tibia, humerus, forearm
Degree of soft tissue damage	0—3
Primary surgery (if applicable)	Date, duration, type of fixation (e.g. cast, external fixator)
Final surgery	Date, duration, type of surgery (external fixator, angular stable plate, LISS plate, screw osteosynthesis, intramedullary nail osteosynthesis, compression plate, neutralization plate, others), usage of adjuvants (BMP-2, platelet rich plasma, autologous cancellous bone graft, allogeneic cancellous bone graft, local application of antibiotics, local muscle flap, remote muscle flap, others)
Blood collection	Date and time of pre- and post-surgery blood collection
**Chronic diseases and medication (FU visits 1–8)**	
Osteoporosis	Calcium, bisphosphonates, Vitamin D, SERM, Strontiumrenalat, Teriparatide, antibody-treatment, phosphate, others
Peripheral arterial disease	Grade, walking length, angioplasty, bypass surgery, thrombendarteriectomy, sympathectomy, vasoactive, prostaglandins, others
Heart failure	NYHA class, type of heart failure, pacemaker, heart valve surgery, bypass surgery, coronary heart disease, percutaneous coronary intervention, acetylsalicylic acid, beta-blocker, angiotensin-I-blocker, ACE inhibitors, diuretics, aldosterone antagonists, loop diuretics, osmotic diuretics, thiazide diuretics, potassium-sparing diuretics, transplantation, others
Arterial hypertension	Acetylsalicylic acid, beta blocker, ACE inhibitors, diuretics, calcium antagonists, angiotensin-I-blocker, others
Chronic obstructive pulmonary disease	GOLD classification, cortisone, PDE-4- blocker, prednisolone, acetylcysteine, others
Tumor disease	Type of tumor, tumor surgery, radiation, chemotherapy, antibody therapy, others
Thyroid disorder	Parathyroidectomy, thyroidectomy, hormone therapy, others
Renal failure	Stadium, Peritoneal dialysis, hemodialysis, hemofiltration, cortisol, transplantation, immunosuppressants,
Diabetes mellitus	Type, conventional insulin therapy, oral antidiabetic drugs, intensive conventional insulin therapy, insulin pump therapy, dietary therapy, combined
Rheumatoid disease	Type, non-steroidal anti-inflammatory drugs, steroidal anti-inflammatory drugs, calcineurin inhibitor, cytostatic drugs, antibody therapy, m-TOR inhibitor
Chronic anemia	Substitution
**Post surgery information (FU visits V4 – V8)**	
Chronic, non-fracture related medication	See “chronic diseases and medication”
Post surgery, fracture related, medication	Usage and duration of vitamin K antagonists, Factor Xa and thrombin inhibitors, acetylsalicylic acid, steroidal anti-inflammatory drugs, non-steroidal anti-inflammatory drugs (NSAID), opioid analgesics
Healing evaluation	X-ray and/or computed tomography, bone consolidated (yes/no), radiographic union scale in tibial fractures (RUST score), radiographic union score for hip (RUSH score), implant set (no/yes; knee joint replacement, hip joint replacement), patient weight-bearing capacity (No load, partial load < 2 kg, 5—10 kg, 10—15 kg, 20 kg, 30 kg, full weight bearing)
X-ray and/or CT images	Storage of the images in eCRF data base
Health economics	Time point of ability to work, payment of sickness benefit, duration and length of physiotherapy units, use of walking aids (armpit supports, walking frame, crutches, rollator), duration of inpatient/partial inpatient stay in a rehabilitation clinic
Patient Reported Outcome Measures	SF-36
Infection status	Pathogen spectrum, medical and/or surgical treatment
Fracture revision treatments	Type and numbers of additive surgical treatment procedures (dynamization, change of procedure within osteosynthesis or to endoprosthesis, use of adjuvants) type and numbers of additive non-surgical fracture treatment procedures (e.g. extended outpatient physiotherapy (EAP), shockwave therapy)
**Adverse events/serious adverse events**	
Diagnosis of AE or SAE:	Type AE´s or SAE´s
Duration of AE/SAE:	Start/end/ongoing
SAE:	Yes/no
Severity:	Mild, moderate, severe
Related to study procedure:	Yes/no/unknown
Intervention:	None/concomitant medication/hospitalization/surgical intervention/other
Outcome:	Recovered/recovered with consequences/ permanent without therapy/permanent with therapy/death/unknown

#### Statistical analyses

An optimal cut-off value for circulating CD8 + TEMRA cell levels (> 38%) was determined in a retrospective pilot study [23]. This predefined threshold, alongside an optimized cut-off identified within the multivariable prediction model, will be used to predict the healing outcome of patients. Based on the final clinical healing classification, sensitivity, specificity and other diagnostic accuracy measures will be calculated for CD8 + TEMRA levels within the covariate-adjusted model. CD8 + TEMRA cells level, together with auxiliary covariates and selected confounders, will be entered into a logistic regression model to predict the occurrence of delayed fracture and non-fracture healing beyond the biomarker alone and/or adjusted. Putative key covariates and confounders are age, sex, diabetes type II, NSAID use, rheumatoid arthritis and osteoporosis. Variable selection will be done using the AIC and validated by using bootstrapping methods. The diagnostic ability of the resulting prediction score will be measured using e.g. Nagelkerkes R2N, Somers Dxy, Brier Score and misclassification error. Further, the score will be analyzed by using AUC/ROC curve and the Youden index. Sensitivity, specificity, NPV, PPV, positive LR and negative LR including 95%-confidence intervals will be calculated. Calibration will be evaluated via calibration plot and goodness-of-fit will be tested using Hosmer–Lemeshow-Test and pseudo-R^2^ measures [30].

For missing data, the proportion of missingness will be assessed. If more than 5% of values are missing, multiple imputation (MI) by chained equations (MICE) will be applied under the assumption that data are Missing Completely at Random (MCAR) or Missing at Random (MAR). The imputation model will include all covariates, outcome variables, and relevant auxiliary variables that may inform the missingness mechanism. A minimum of 20 imputations will be generated. To assess the robustness of the results, sensitivity analyses will be conducted by comparing the multiply imputed datasets with complete-case analyses. Additional variables will be analyzed using appropriate parametric or non-parametric statistical tests, depending on scale and distribution. All p-values from secondary analyses will be interpreted in an exploratory, non-confirmatory manner. All statistical analyses will be done using SAS (version 9.4) and R (latest version at time of analysis).

#### Outcome measures and clinical endpoints

Normal fracture healing typically occurs within 18 weeks, while non-union is usually diagnosed beyond 6 months after the initial fracture. Although no standardized clinical guideline exists for the precise timing of complete fracture consolidation, international studies support the proposed study endpoints and visits schedule for monitoring the fracture healing process [14, 34, 35]. The following classification criteria have been defined:

**Table:** 

Classification	Time post-surgery	Classification criteria
Normal healing	≤ 5 month	Fracture consolidation
Delayed healing	> 5 month and < 9 month	Fracture consolidation or Additional supportive non-invasive treatment or Additive (supportive) surgical treatment
Non-union	> 9–12 month	No fracture consolidation or Additive (supportive) surgical treatment

Incomplete consolidation is defined by one or more of the following criteria: a) presence of resorption zone or incomplete callus formation, b) incomplete bridging (1–3 cortices), or c) no bridging (no cortex is bridged).

Radiographic evidence of bony bridging or complete filling of the fracture gap will serve as the gold standard for biomarker validation.

Radiological signs of bony consolidation are: a) fracture is continuously bridged, b) fracture callus shows homogeneous density, and c) density of the fracture callus is comparable to the cortical density. These signs must be detectable in at least 2 planes. Therefore, the evaluation of the postoperative X-ray images in 2 planes will be used to assess the bony consolidation. If the findings at the primary and/or secondary endpoints are unclear by conventional radiological analysis, the additional performance of a CT scan is medically justifiable and indicated. Furthermore, the RUSH and RUST score will be used to quantify the radiological imaging. Based on the evaluation of the cortices, a RUST score > 10 or a RUSH score > 24 results in consolidation [36–41] (Table 3).

**Table 3:** Primary-, secondary- and exploratory endpoints

**Primary Efficacy Endpoint:**
• Successful fracture consolidation at V6 (week 17–19) based on clinical and radiological assessment
**Secondary Efficacy Endpoints:**
1. Successful fracture consolidation at V7 (week 34–36) based on clinical and radiological assessment 2. Time point ability to work 3. RUST/RUSH score at V6, V7, V8
**Exploratory Endpoints:**
1. Proportion of subjects with an additional intervention independent of a surgical or non-surgical treatment to foster the fracture healing process 2. Duration of physiotherapy sessions done with a professional within 17–19 weeks post-surgery 3. Proportion of subjects with complete weight bearing within 17–19 weeks post surgery 4. Proportion of subjects with inpatient or day-care rehabilitation within 34–36 weeks post surgery

### Data management and data monitoring

All collected data will be entered by the on-site study staff into secuTrial system, an electronic data capture system that is compliant to the Food and Drug Administration (FDA) 21 CFR Part 11 and ICH-GCP, and automatically keeps an audit trail of all entries and corrections to the electronic case report forms (eCRFs). Data will be monitored onsite as well as centrally by the Clinical Study Center—Charité – Universitätsmedizin Berlin (KKS) to ensure its accuracy and integrity. After the last study patient will have been completed the study and all data has been entered and checked for completeness and plausibility, the database will be closed. The KKS will then create and transmit the final database.

### Trial organization and oversight

The BioBone study is funded by the Federal Ministry of Education and Research (BMBF grant number 01EK1514). The BioBone consortium is coordinated by Charité – Universitätsmedizin Berlin and includes the partner institutions (see Acknowledgements).

Yearly meetings are held during which all partners gather to discuss study status, problems, and solutions in the consortium-wide forum and to ensure annual progress report for the BMBF.

## Discussion

There is a clear medical need for early prediction and prevention of delayed or incomplete fracture healing, which is a significant medical concern in industrialized countries. The validation of circulating CD8 + TEMRA levels as a biomarker for fracture healing aims to provide novel prognostic options for treatment strategies to avoid insufficient clinical healing outcomes. An early detection of impaired fracture healing may lead to better mobility, quality of life of our patients and less revision operations. Even more proactive bone healing support and specific osteosynthesis techniques may be applied to these patients in the future. Therefore, a successful integration of this biomarker into clinical practice could facilitate personalized therapeutic approaches, enabling early identification of patients at risk and guiding therapeutic decisions, such as tailored surgical strategies, including the application of biologics, and individualized rehabilitation protocols. It may also foster the development of immune-modulatory therapies to improve endogenous bone repair.

## Data Availability

No datasets were generated or analysed during the current study.

## References

[1] Collaborators GBDF. Global, regional, and national burden of bone fractures in 204 countries and territories, 1990–2019: a systematic analysis from the Global Burden of Disease Study 2019. Lancet Healthy Longev. 2021;2(9):e580–92. 34723233 10.1016/S2666-7568(21)00172-0 PMC8547262

[2] Panteli M, Vun JSH, Pountos I. A JH, Jones E, Giannoudis PV: Biological and molecular profile of fracture non-union tissue: A systematic review and an update on current insights. J Cell Mol Med. 2022;26(3):601–23. 34984803 10.1111/jcmm.17096 PMC8817135

[3] Pountos I, Georgouli T, Pneumaticos S, Giannoudis PV. Fracture non-union: Can biomarkers predict outcome? Injury. 2013;44(12):1725–32. 24075219 10.1016/j.injury.2013.09.009

[4] Wildemann B, Ignatius A, Leung F, Taitsman LA, Smith RM, Pesantez R, Stoddart MJ, Richards RG, Jupiter JB. Non-union bone fractures. Nat Rev Dis Primers. 2021;7(1):57. 34354083 10.1038/s41572-021-00289-8

[5] Calori GM, Tagliabue L, Gala L, d’Imporzano M, Peretti G, Albisetti W. Application of rhBMP-7 and platelet-rich plasma in the treatment of long bone non-unions: a prospective randomised clinical study on 120 patients. Injury. 2008;39(12):1391–402. 19027898 10.1016/j.injury.2008.08.011

[6] Kanakaris NK, Lasanianos N, Calori GM, Verdonk R, Blokhuis TJ, Cherubino P, De Biase P, Giannoudis PV. Application of bone morphogenetic proteins to femoral non-unions: a 4-year multicentre experience. Injury. 2009;40(Suppl 3):S54-61. 20082793 10.1016/S0020-1383(09)70013-0

[7] Kanakaris NK, Calori GM, Verdonk R, Burssens P, De Biase P, Capanna R, Vangosa LB, Cherubino P, Baldo F, Ristiniemi J, et al. Application of BMP-7 to tibial non-unions: a 3-year multicenter experience. Injury. 2008;39(Suppl 2):S83-90. 18804578 10.1016/S0020-1383(08)70019-6

[8] Ekegren CL, Edwards ER, de Steiger R, Gabbe BJ: Incidence, Costs and Predictors of Non-Union, Delayed Union and Mal-Union Following Long Bone Fracture. Int J Environ Res Public Health. 2018;15(12):2845. 10.3390/ijerph15122845. 10.3390/ijerph15122845 PMC6313538 30551632

[9] Vanderkarr MF, Ruppenkamp JW, Vanderkarr M, Holy CE, Blauth M. Risk factors and healthcare costs associated with long bone fracture non-union: a retrospective US claims database analysis. J Orthop Surg Res. 2023;18(1):745. 37784206 10.1186/s13018-023-04232-3 PMC10546674

[10] Alt V, Donell ST, Chhabra A, Bentley A, Eicher A, Schnettler R. A health economic analysis of the use of rhBMP-2 in Gustilo-Anderson grade III open tibial fractures for the UK, Germany, and France. Injury. 2009;40(12):1269–75. 19539926 10.1016/j.injury.2009.02.007

[11] Ji X, Zhao D, Xin Z, Feng H, Huang Z. The predictive value of stress-induced hyperglycemia parameters for delayed healing after tibial fracture post-surgery. J Orthop Surg Res. 2024;19(1):666. 39415173 10.1186/s13018-024-05138-4 PMC11484393

[12] Duda GN, Geissler S, Checa S, Tsitsilonis S, Petersen A, Schmidt-Bleek K. The decisive early phase of bone regeneration. Nat Rev Rheumatol. 2023;19(2):78–95. 36624263 10.1038/s41584-022-00887-0

[13] Tsegaye YA, Tegegne BB, Ayehu GW, Amisalu BT, Sulala AC. Prospective study on functional outcome of distal femur fracture treated by open reduction and internal fixation using distal femur locking plate in Tibebe Ghion Specialized Hospital, Bahirdar, North West Ethiopia. J Orthop Surg Res. 2024;19(1):582. 39304870 10.1186/s13018-024-05054-7 PMC11414311

[14] Gelalis ID, Politis AN, Arnaoutoglou CM, Korompilias AV, Pakos EE, Vekris MD, Karageorgos A, Xenakis TA. Diagnostic and treatment modalities in nonunions of the femoral shaft: a review. Injury. 2012;43(7):980–8. 21741650 10.1016/j.injury.2011.06.030

[15] Cao X, Tang Q, Zhou B, Xiao W, Chen H. Comparison of the efficacy of intramedullary nailing via the lateral parapatellar approach versus the infrapatellar approach in the treatment of tibial metaphyseal-diaphyseal junction fractures. J Orthop Surg Res. 2024;19(1):838. 39695731 10.1186/s13018-024-05338-y PMC11656785

[16] Nicholson JA, Yapp LZ, Keating JF, Simpson A. Monitoring of fracture healing Update on current and future imaging modalities to predict union. Injury. 2021;52 Suppl 2:S29–34. 32826052 10.1016/j.injury.2020.08.016

[17] Migliorini F, Cocconi F, Vecchio G, Schaefer L, Koettnitz J, Maffulli N. Pharmacological agents for bone fracture healing: talking points from recent clinical trials. Expert Opin Investig Drugs. 2023;32(9):855–65. 37740660 10.1080/13543784.2023.2263352

[18] de Martinez Albornoz P, Khanna A, Longo UG, Forriol F, Maffulli N. The evidence of low-intensity pulsed ultrasound for in vitro, animal and human fracture healing. Br Med Bull. 2011;100:39–57. 21429948 10.1093/bmb/ldr006

[19] Bucher CH, Schlundt C, Wulsten D, Sass FA, Wendler S, Ellinghaus A, Thiele T, Seemann R, Willie BM, Volk HD, et al. Experience in the Adaptive Immunity Impacts Bone Homeostasis, Remodeling, and Healing. Front Immunol. 2019;10:797. 31031773 10.3389/fimmu.2019.00797 PMC6474158

[20] El Khassawna T, Serra A, Bucher CH, Petersen A, Schlundt C, Konnecke I, Malhan D, Wendler S, Schell H, Volk HD, et al. T Lymphocytes Influence the Mineralization Process of Bone. Front Immunol. 2017;8:562. 28596766 10.3389/fimmu.2017.00562 PMC5442173

[21] Schmidt-Bleek K, Schell H, Lienau J, Schulz N, Hoff P, Pfaff M, Schmidt G, Martin C, Perka C, Buttgereit F, et al. Initial immune reaction and angiogenesis in bone healing. J Tissue Eng Regen Med. 2014;8(2):120–30. 22495762 10.1002/term.1505

[22] Toben D, Schroeder I, El Khassawna T, Mehta M, Hoffmann JE, Frisch JT, Schell H, Lienau J, Serra A, Radbruch A, et al. Fracture healing is accelerated in the absence of the adaptive immune system. J Bone Miner Res. 2011;26(1):113–24. 20641004 10.1002/jbmr.185

[23] Reinke S, Geissler S, Taylor WR, Schmidt-Bleek K, Juelke K, Schwachmeyer V, Dahne M, Hartwig T, Akyuz L, Meisel C, et al. Terminally differentiated CD8(+) T cells negatively affect bone regeneration in humans. Sci Transl Med. 2013;5(177):177ra136. 10.1126/scitranslmed.3004754 23515078

[24] Schlundt C, Reinke S, Geissler S, Bucher CH, Giannini C, Mardian S, Dahne M, Kleber C, Samans B, Baron U, et al. Individual Effector/Regulator T Cell Ratios Impact Bone Regeneration. Front Immunol. 1954;2019:10. 10.3389/fimmu.2019.01954 PMC6706871 31475013

[25] Voss JO, Pivetta F, Elkilany A, Schmidt-Bleek K, Duda GN, Odaka K, Dimitriou IM, Ort MJ, Streitz M, Heiland M, et al. Prognostic implications of a CD8(+) T(EMRA) to CD4(+)T(reg) imbalance in mandibular fracture healing: a prospective analysis of immune profiles. Front Immunol. 2024;15:1476009. 39507538 10.3389/fimmu.2024.1476009 PMC11537918

[26] Hoff P, Gaber T, Strehl C, Schmidt-Bleek K, Lang A, Huscher D, Burmester GR, Schmidmaier G, Perka C, Duda GN, et al. Immunological characterization of the early human fracture hematoma. Immunol Res. 2016;64(5–6):1195–206. 27629117 10.1007/s12026-016-8868-9

[27] Schlundt C, Schell H, Goodman SB, Vunjak-Novakovic G, Duda GN, Schmidt-Bleek K. Immune modulation as a therapeutic strategy in bone regeneration. J Exp Orthop. 2015;2(1):1. 26914869 10.1186/s40634-014-0017-6 PMC4545842

[28] Elazaly H, Dimitriou IM, Maleitzke T, Dahne M, Jaecker V, Maerdian S, Tafelski S, Diekhoff T, Lindner T, Akgun D, et al. ILOBONE: A phase I/IIa randomized controlled trial to assess the safety and feasibility of local iloprost therapy for enhancing proximal humerus fracture healing- a pilot study design. J Orthop Surg Res. 2025;20(1):498. 40405317 10.1186/s13018-025-05865-2 PMC12096472

[29] Krummenauer F, Kauczor HU. Sample size determination in reference-controlled diagnostic trials. Rofo. 2002;174(11):1438–44. 12424672 10.1055/s-2002-35346

[30] Harrell FE: Regression Modeling Strategies: With Applications to Linear Models, Logistic Regression, and Survival Analysis: Springer-Verlag; 2001.

[31] Kverneland AH, Streitz M, Geissler E, Hutchinson J, Vogt K, Boes D, Niemann N, Pedersen AE, Schlickeiser S, Sawitzki B. Age and gender leucocytes variances and references values generated using the standardized ONE-Study protocol. Cytometry A. 2016;89(6):543–64. 27144459 10.1002/cyto.a.22855

[32] Sawitzki B, Harden PN, Reinke P, Moreau A, Hutchinson JA, Game DS, Tang Q, Guinan EC, Battaglia M, Burlingham WJ, et al. Regulatory cell therapy in kidney transplantation (The ONE Study): a harmonised design and analysis of seven non-randomised, single-arm, phase 1/2A trials. Lancet. 2020;395(10237):1627–39. 32446407 10.1016/S0140-6736(20)30167-7 PMC7613154

[33] Streitz M, Miloud T, Kapinsky M, Reed MR, Magari R, Geissler EK, Hutchinson JA, Vogt K, Schlickeiser S, Kverneland AH, et al. Standardization of whole blood immune phenotype monitoring for clinical trials: panels and methods from the ONE study. Transplant Res. 2013;2(1):17. 24160259 10.1186/2047-1440-2-17 PMC3827923

[34] Calori GM, Mazza EL, Mazzola S, Colombo A, Giardina F, Romano F, Colombo M. Non-unions. Clin Cases Miner Bone Metab. 2017;14(2):186–8. 29263731 10.11138/ccmbm/2017.14.1.186 PMC5726207

[35] Guidance document for the preparation of investigational device exemptions and pre-market approval applications for bone growth stimulator devices [https://www.fda.gov/ohrms/dockets/98fr/98d0238].

[36] Kizkapan TB, Misir A, Oguzkaya S, Ozcamdalli M, Uzun E, Sayer G. Reliability of radiographic union scale in tibial fractures and modified radiographic union scale in tibial fractures scores in the evaluation of pediatric forearm fracture union. Jt Dis Relat Surg. 2021;32(1):185–91. 33463435 10.5606/ehc.2021.78465 PMC8073431

[37] Zhou TJ, Jiang S, Ren JK, Zhang X, Liu WX, Yan P, Li JW, Zeng T, Xu ZS. Improving agreement in assessing subtrochanteric fracture healing among orthopedic surgeons using the Radiographic Union Score for Hip (RUSH). BMC Musculoskelet Disord. 2024;25(1):798. 39385152 10.1186/s12891-024-07902-3 PMC11463057

[38] Chandran M, Akesson KE, Javaid MK, Harvey N, Blank RD, Brandi ML, Chevalley T, Cinelli P, Cooper C, Lems W, et al. Impact of osteoporosis and osteoporosis medications on fracture healing: a narrative review. Osteoporos Int. 2024;35(8):1337–58. 38587674 10.1007/s00198-024-07059-8 PMC11282157

[39] Frank T, Osterhoff G, Sprague S, Garibaldi A, Bhandari M, Slobogean GP, Investigators F. The Radiographic Union Score for Hip (RUSH) Identifies Radiographic Nonunion of Femoral Neck Fractures. Clin Orthop Relat Res. 2016;474(6):1396–404. 26728521 10.1007/s11999-015-4680-4 PMC4868173

[40] Graf R, Shaw J, Simske N, Siy P, Siy A, Kliethermes S, Whiting P. Distal femur nonunion: Risk factors and validation of RUST scores. OTA International: The Open Access Journal of Orthopaedic Trauma. 2023;6(1):e234.

[41] Bhandari M, Chiavaras MM, Parasu N, Choudur H, Ayeni O, Chakravertty R, Bains S, Hak A, Sprague S, Petrisor B. Radiographic union score for hip substantially improves agreement between surgeons and radiologists. BMC Musculoskelet Disord. 2013;14:70. 23442540 10.1186/1471-2474-14-70 PMC3599458

